# Editorial: New trends in distributed and autonomous intelligent systems for crop production

**DOI:** 10.3389/fpls.2026.1970194

**Published:** 2026-09-22

**Authors:** Xu Zheng

**Affiliations:** School of Computer Science and Engineering, University of Electronic Science and Technology of China, Chengdu, China

**Keywords:** artificial intelligence, autonomous intelligence, computer vision, knowledge graph, large language model

The goal of the Research Topic entitled “New Trends in Distributed and Autonomous Intelligent Systems for Crop Production” is to inspire and collect innovative and high-quality studies addressing fundamental challenges in building distributed, autonomous, and intelligent systems for modern crop production. This Research Topic was published on 2025.05.28 and due on 2026.03.30. During the whole process, the Research Topic has received a total of 13 submissions, among which 6 outstanding papers are accepted. These papers have contributed to the development of distributed and autonomous intelligent systems for crop production by considering the design of lightweight artificial intelligence (AI) models, edge computing paradigms, knowledge-driven reasoning, and large language model (LLM)-empowered decision-making, and have shown great potential in benefiting various applications like sugarcane node detection, garlic damage detection, olive fruit detection, litchi segmentation, plant knowledge graph reasoning, and intelligent plant protection. Some highlights of the Research Topic are summarized as follow.

The Research Topic has inspired studies on new results on lightweight computer vision (CV) models and edge deployment strategies for crop production. With the proliferation of unmanned aerial vehicles (UAVs), autonomous harvesters, and Internet-of-Things (IoT) devices in agriculture, the demand for efficient, accurate, and deployable perception models at the edge has become increasingly urgent. The Research Topic has published four outstanding papers dealing with the design of lightweight deep learning models for different crop production tasks, including sugarcane node detection, garlic damage detection, olive fruit detection, and clustered litchi segmentation. These studies have considered the most advanced models like YOLOv8, YOLOv11n, and U-Net, and provided refined designs to adapt the models for resource-constrained edge devices and complex field environments. Specifically, all authors have conducted extensive experiments to validate the improved performance. For example, the model EdgeSugarcane proposed in the paper “EdgeSugarcane: A Lightweight High-Precision Method for Real-Time Sugarcane Node Detection in Edge Computing Environments” achieved a precision of 93.5%, a recall of 80.0%, and a mAP of 87.0% on the test set, with the inference time reduced to only 1.9 ms after TensorRT-based FP16 quantization, which is 3.3 times faster than before optimization. Meanwhile, the Garlic-YOLO-DD model proposed in “Garlic-YOLO-DD: A Lightweight Object Detection Algorithm for Garlic Damage Detection” reduced the number of parameters to 57.96% of YOLOv11n, decreased computational load by 20.63%, increased inference speed by 15.97%, and improved mAP@50 by 27.64 percentage points. The YOLO-TinyFuse model in “A Lightweight YOLO-TinyFuse Model for Small Target Detection of Olive Fruits” achieved an mAP50 of 92.3% and a recall of 84.5%, outperforming YOLOv8n by 2.6% and 3.2%, respectively, while reducing parameters by 6.76%. Furthermore, the DSD-UNet model in “A Lightweight DSD-UNet-Based Method for Clustered Litchi Segmentation” achieved an mIoU of 91.40% and an F1-score of 95.42%, with inference speed increased by 138.9% and parameter count reduced by 61.5% compared with the original U-Net. Generally, these studies extended the design of lightweight CV models and edge deployment strategies for more robust and efficient autonomous systems in crop production.

Besides, our Research Topic also collected impressive studies on intelligent decision-making and knowledge-driven systems for crop production. The first study, entitled “Research on Plant Knowledge Graph Reasoning Based on Dual-Channel Attention and Topological Perception,” proposed the KRGAI-PLANT model, which addressed the challenge of long-range dependency reasoning in plant knowledge graphs by integrating a global attention mechanism with local topological perception. This work provided an effective reasoning tool for transforming multi-source plant data into actionable knowledge, supporting association prediction and decision-making in precision agriculture, ecological monitoring, and intelligent plant protection. The second study, entitled “Challenges and Strategies for Harnessing Large Language Models in Plant Protection,” provided a comprehensive perspective on the opportunities and challenges of applying LLMs in plant protection. The authors discussed key application areas such as pest and disease monitoring, control decision support, plant quarantine risk assessment, and pesticide development, and proposed strategies including domain-specific fine-tuning, knowledge-graph grounding, GraphRAG, multi-agent collaboration, model compression, and hierarchical edge-cloud deployment. These studies have set fundamental supports for building distributed and autonomous intelligent systems in crop production, ranging from structured knowledge reasoning to natural language-based decision support.

Generally, the Research Topic has provided a forum sharing pioneering ideas and investigations on distributed and autonomous intelligent systems for crop production, and collected a batch of AI models and frameworks specifically tailored for intelligent crop production. During the whole process, the Research Topic received 2,165 downloads, 7,708 article views and 12K topic views. I believe that the Research Topic and these accepted papers will shed light on the subsequent studies on cutting-edge techniques for distributed and autonomous intelligent systems in crop production.

